# Advantages of using reduced-volume intensity modulated radiation therapy for the treatment of nasopharyngeal carcinoma: a retrospective paired study

**DOI:** 10.1186/s12885-019-5774-2

**Published:** 2019-06-08

**Authors:** Fang Liu, Ting Luo, Tao Jin, Jiahui Zhang, Zhongzheng Xiang, Ruonan Yan, Li Xie, Xin Wu, Hong Zhang, Feng Wang, Ping Li, Lei Liu

**Affiliations:** 1 Department of Radiation Oncology, Cancer Center, State Key Laboratory of Biotherapy, West China Hospital, Sichuan University, Chengdu, 610041 Sichuan China; 2 Mammary Oncology and Department of Medical Oncology, Clinical Research Center for Breast, Cancer Center, Laboratory of Molecular Diagnosis of Cancer, West China Hospital, Sichuan University, Chengdu, 610041 Sichuan China; 3 Cancer Center, State Key Laboratory of Biotherapy, West China Hospital, Sichuan University, Chengdu, 610041 Sichuan China

**Keywords:** Nasopharyngeal carcinoma, Radiotherapy, Intensity modulated radiation therapy, Clinical target volume

## Abstract

**Background:**

The definition of clinical target volume (CTV) in intensity modulated radiation therapy (IMRT) for nasopharyngeal carcinoma (NPC) has not been addressed. We performed this study to assess the feasibility and efficacy of using IMRT with reduced-volume CTV for the treatment of nasopharyngeal carcinoma.

**Methods:**

We retrospectively reviewed 293 non-metastatic NPC patients treated with IMRT from 2002 to 2013. A total of 180 matched cases finally included with 90 received conventional-volume IMRT (CV-IMRT) and 90 received reduced-volume IMRT (RV-IMRT). Kaplan-Meier method and log-rank tests were used to compare NPC-specific survival. Multivariate analyses using the Cox proportional hazards model were conducted to detect independent predictors.

**Results:**

With a median follow-up of 70 months, the 3-year overall survival, progression-free survival, distant metastasis-free survival, local recurrence-free survival, regional recurrence-free survival, locoregional recurrence-free survival rates were 88.9, 84.4, 92.2, 91.1, 98.9, 91.1% for the CV-IMRT arm and 92.2, 85.6, 90.0, 93.3, 98.9, 93.3% for the RV-IMRT arm, respectively. None significant survival difference was observed. Additionally, RV-IMRT was associated with reduced risk of late xerostomia (*P* = 0.039) and hearing loss (*P* = 0.008), compared versus CV-IMRT.

**Conclusions:**

The use of RV-IMRT for the treatment of NPC led to comparable survival condition and truly reduced toxicity reactions compared versus CV-IMRT.

## Background

Nasopharyngeal carcinoma (NPC) is a malignancy that shows high prevalence in Southeast Asia and Southern China [1, 2]. Radiotherapy has been regarded as the most effective and only curative treatment modality for NPC [3]. The locoregional control of NPC chiefly depends on high-dose radiotherapy, whereas the adjacent critical, dose-limiting normal structures are inevitably exposed to rays. Intensity modulated radiation therapy (IMRT), a way of breaking through in the treatment of NPC, offering significant advantages in target dose uniformity and the sparing of adjacent organs at risk, causing a more satisfactory disease control and a lower rate of toxicity than traditional radiotherapy [4–6]. A number of studies [7–10] have constantly reported an over 85% local control of NPC treated with IMRT. Although IMRT has been widely used, the selection and definition of clinical target volume (CTV) in IMRT for NPC has not yet reached a consensus. The Radiation Therapy Oncology Group (RTOG) trials involving IMRT of NPC put forward the delineation of CTV [11, 12], which were largely based on our previous experience in two-dimensional radiotherapy (2D-RT). According to the previous research results, locoregional recurrence has become a main failure mode and the majority occurs within the treatment field. Nevertheless, isolated marginal recurrence was hardly found even in advanced T stage cases [13–15], which signified that the wide field used in 2D-RT might be redundant. In addition, increased high-dose treatment volumes almost surely increase radiation toxicity reactions, as well as the incidence of second primary tumor (SPT). The intentional coverage of all adjacent structures in CTV may be unnecessary and disputable, especially in IMRT era. Based on these, Fujian Provincial Tumor Hospital Affiliated to Fujian Medical University formulated a reduced-volume IMRT (RV-IMRT) delineation [16]. To our knowledge, there has not been direct comparison between the two protocols in the treatment of NPC.

We performed this study to investigate the feasibility and efficacy of using IMRT with reduced-volume CTV for the treatment of NPC, with the hope to provide certain reference meanings for further studies.

## Methods

### Patients

Between December 2002 and August 2013, 293 histologically diagnosed, non-metastatic and treatment-naive nasopharyngeal carcinoma patients were treated with IMRT at our center. Pretreatment staging evaluation consisted of a complete physical examination, liver and renal biochemical analysis, complete blood cell count, flexible fiberoptic endoscopic examination, computed tomography (CT) scanning/magnetic resonance imaging (MRI) of the head and neck, bone scan, chest X-ray, ultrasonography of the abdomen, and dental evaluation. Positron emission tomography scans and CT scans of the chest/abdomen were performed when necessary. Tumors were staged according to the American Joint Committee on Cancer (AJCC) 2010 cancer staging classification.

### Radiotherapy

Between December 2002 and December 2009, the first 122 NPC patients were treated with IMRT and the target volumes were delineated by taking RTOG protocol as the reference [11], which was regarded as the conventional-volume IMRT (CV-IMRT) group. As determined by clinical, endoscopic examinations and imaging, the gross tumor volume (GTV) included the primary nasopharyngeal tumor (GTV-P) and the involved lymph nodes (GTV-N). The clinical target volume was defined as the subclinical regions at risk for involvement. The CTV-1 was defined as GTV plus areas at risk including the entire nasopharynx, skull base, parapharyngeal space, retropharyngeal space, pterygopalatine fossae, clivus, the inferior part of the sphenoid sinus, the posterior third of the nasal cavity and maxillary sinuses, and the upper deep jugular nodes. The CTV-2 was contoured as middle and lower jugular nodes. The planning target volumes (PTVs) were expanded by 3 mm from the above volumes in case of set-up uncertainties and kinematic errors. Generally, a total dose of 70 Gy in 33 fractions at 2.12 Gy/fraction to the PTVs of GTV-P and GTV-N, 60 Gy in 33 fractions at 1.82 Gy/fraction to the PTV of CTV-1, 56 Gy in 33 fractions at 1.70 Gy/fraction to the PTV of CTV-2 were prescribed.

The reduced-volume IMRT (RV-IMRT) group enrolled the next 171 NPC patients during January 2010 to August 2013. The GTV was defined as that described for the CV-IMRT arm. The CTV-1 was contoured as GTV plus 5-10 mm margin, as well as the nasopharyngeal mucosa plus 5 mm submucosal region. The CTV-2 covered areas including the nasopharyngeal cavity (the posterior part of nasal cavity), parapharyngeal space, maxillary sinus (the anterior 5 mm part of the posterior nasal aperture and maxillary mucosa), posterior ethmoid sinus, the inferior part of spheniod sinus and cavernous sinus, skull base, the anterior third part of clivus and cervical vertebra, pterygopalatine fossa, and retropharyngeal lymph nodes (from skull base to cranial edge of the C2 vertebra). The CTV-N included levels II to V nodal regions (upper deep jugular nodes were not covered unless involved). The PTVs were expanded by 3 mm from the above volumes. A total dose of 70 Gy in 33 fractions at 2.12 Gy/fraction to the PTVs of GTV-P and GTV-N, 60 Gy in 33 fractions at 1.82 Gy/fraction to the PTV of CTV-1, 56 Gy in 33 fractions at 1.70 Gy/fraction to the PTVs of CTV-2 and CTV-N were prescribed.

No matter what kind of radiation protocol was used, critical normal structures including the spinal cord, brainstem, temporal lobes, hypophysis, optic nerves, chiasm, eyeballs, lens, parotid glands, temporomandibular joints and mandible were set as organs at risk (OARs). The dose received by each OAR was limited according to the RTOG protocol. At our center, computerized optimization was utilized with fusion of MRI with planning CT images to accurately delineate the target volumes. Treatment plans were generated using the Elekta PrecisePLAN (Release 2.10). Patients received IMRT with 6-MV X-ray beams modulated using Elekta Precise and Elekta Synergy VMAT (Elekta, Stockholm, Sweden). In general, treatment was delivered one fraction daily, 5 days per week.

### Chemotherapy

Patients from both groups presented with stage II-IVb were treated with IMRT combined with cisplatin-based concurrent chemotherapy (cisplatin 80 mg/m^2^ divided into 3 parts on days 1–3, every 3 weeks). Neoadjuvant or adjuvant chemotherapy was given at discretion of the attending physician, chemotherapy protocols including PF (cisplatin 80 mg/m^2^ divided into 3 parts on days 1–3, and fluorouracil 750 mg/m^2^ per day on days 1–5, every 3 weeks) and TPF (paclitaxel 135 mg/m^2^ day 1, cisplatin 80 mg/m^2^ divided into 3 parts on days 1–3, and fluorouracil 750 mg/m^2^ per day on days 1–5, every 3 weeks).

### Follow-up

All patients were required to be followed up after the completion of treatment: every 3 months in the first 2 years, every 6 months over the following 3 years, and then annually thereafter. Each follow-up consisted of physical examination, flexible fiberoptic endoscopy, ultrasound of abdomen, chest X-ray, and basic serum chemistry. Either CT or MRI scans of the head and neck were performed after the completion of radiotherapy and then when clinically needed to evaluate the treatment response. Late toxicities were defined as symptoms occurred beyond 3 months after the completion of treatment and were assessed at each follow-up according to the Common Terminology Criteria for Adverse Events (CTCAE v4.0) [17].

### Statistical analysis

To minish the interference of heterogeneity, patients were paired using propensity score matching (PSM) method [18]. Propensity scores for each patient were computed based on the following covariates: sex, age, T-stage, N-stage, clinical stage, use of chemotherapy (concurrent chemotherapy, neoadjuvant or adjuvant chemotherapy). Patients were then matched at the ratio of 1:1 to create similar case and control arms with balanced characteristics. The balance between the two arms was examined by chi-square test or rank sum test (ranked data).

In this study, overall survival (OS), progression-free survival (PFS), distant metastasis-free survival (DMFS), local recurrence-free survival (LRFS), regional recurrence-free survival (RRFS) and locoregional recurrence-free survival (LRRFS) were analyzed as the endpoints. The duration of time to recurrence and distant metastasis was counted from the completion of radiotherapy until treatment failure. The duration of OS was measured from diagnosis until death or until the last follow-up for patients still alive. For locoregional recurrence cases, in-field failure was determined as 95% or more of the recurrence volume within the 95% isodose. Marginal failure was defined as 20 to 95% of the recurrence volume within the 95% isodose. Out-field failure was defined as less than 20% of the recurrence volume within the 95% isodose. OS, PFS, DMFS, LRFS, RRFS and LRRFS were estimated using the Kaplan-Meier method [19] and pairwise comparisons between groups were calculated using log-rank tests. To detect independent predictors, multivariate analyses were performed using the Cox proportional hazards model. Rank sum test was adopted to compare the adverse events.

All statistical analyses were conducted using IBM SPSS Statistics version 23.0. All tests were two sided, and a *P* value of less than 0.05 was considered statistically significant.

## Results

### Patient clinical characteristics

Of the 293 untreated non-metastatic NPC patients, 180 paired cases finally included with 90 received CV-IMRT and 90 received RV-IMRT. There were 12 (6.7%), 32 (17.8%), 83 (46.1%), and 53 (29.4%) patients presented with stage I, II, III and IV, respectively. Additionally, 164 (91.1%) received concurrent chemotherapy, and 163 (90.6%) received neoadjuvant or adjuvant chemotherapy. The matched cases in both groups had balanced characteristics (all *P* > 0.05). Table 1 summarized the clinical characteristics of the two arms.

**Table 1:** Patient clinical characteristics

Characteristic	CV-IMRT	RV-IMRT	*P* value
	*N* = 90	*N* = 90	
Gender			0.864
Male	66	68	
Female	24	22	
Age			1
< 45	40	40	
≥ 45	50	50	
T stage			0.976
T1	20	23	
T2	32	27	
T3	17	18	
T4	21	22	
N stage			0.889
N0	13	12	
N1	25	26	
N2	45	44	
N3	7	8	
Clinical stage			0.917
I	6	6	
II	16	16	
III	42	41	
IV	26	27	
Concurrent chemotherapy			0.794
No	9	7	
Yes	81	83	
Neoadjuvant/adjuvant chemotherapy			1
No	9	8	
Yes	81	82	

*CV-IMRT* conventional-volume intensity-modulated radiation therapy, *RV-IMRT* reduced-volume intensity-modulated radiation therapy

### Survival outcomes

The median follow-up time was 70 months (range, 10–166 months) for the entire population, 107 months (range, 10–166 months) for the CV-IMRT arm and 50 months (range, 11–78 months) for the RV-IMRT arm, respectively. At the time of the last follow-up, 17 (9.4%), 21 (11.7%) and 35 (19.4%) cases had developed locoregional recurrence, distant metastasis and disease progress, respectively. Three patients had developed both distant metastasis and recurrence. Eleven (12.2%) cases of locoregional failure occurred in the CV-IMRT group with ten failed only in nasopharynx and one failed both in nasopharynx and regional nodal. In the RV-IMRT arm, five cases failed only in nasopharynx, and one failed both in nasopharynx and regional nodal. Of the total 17 locoregional failures, most cases (76.5%) were in-field failures. With regard to the 21 distant metastasis cases, six patients had multiorgan metastasis, and 15 developed metastasis in an organ: 7 cases in lung, 4 cases in bone. Table 2 summarized the failure patterns.

**Table 2:** Failure patterns in the 180 patients after treatment

Patterns of failure	CV-IMRT n (%)	RV-IMRT n (%)
Recurrence	11 (12.2%)	6 (6.7%)
NP recurrence only	10 (11.1%)	5 (5.6%)
LN regions recurrence only	0	0
NP and LN regions recurrence	1 (1.1%)	1 (1.1%)
In-field failures	8 (8.9%)	5 (5.6%)
Marginal failures	1 (1.1%)	1 (1.1%)
Out-field failures	2 (2.2%)	0
Distant metastasis	10 (11.1%)	11 (12.2%)
Distant metastasis and recurrence	1 (1.1%)	2 (2.2%)

*CV-IMRT* conventional-volume intensity-modulated radiation therapy, *RV-IMRT* reduced-volume intensity-modulated radiation therapy, *NP* nasopharynx, *LN* lymph node

**Fig. 1:**
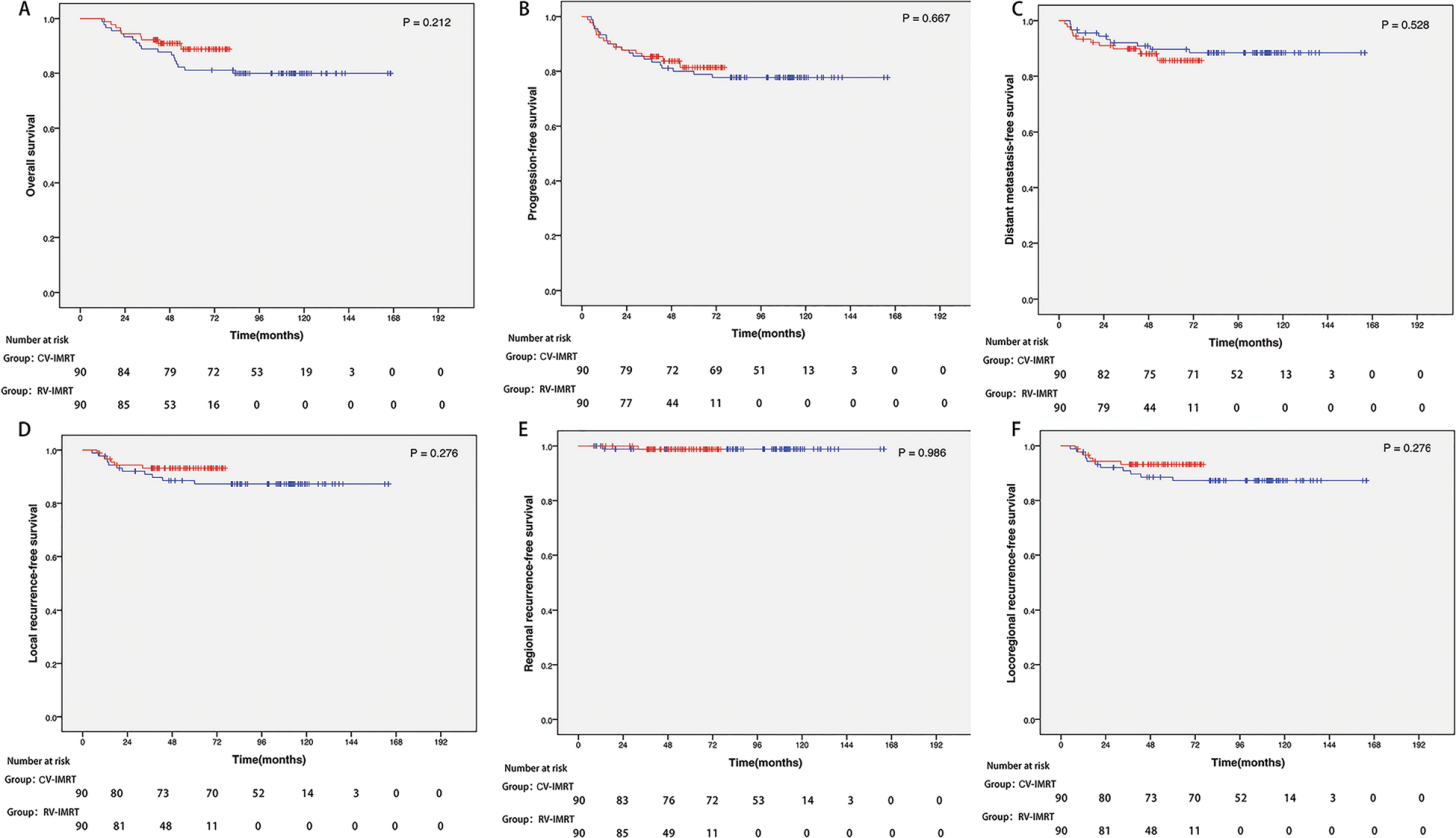
Kaplan-Meier curves illustrate the survival of patients treated with IMRT. Conventional-volume intensity-modulated radiation therapy (blue line) and reduced-volume intensity-modulated radiation therapy (red line), including (**a**) overall survival, (**b**) progression-free survival, (**c**) distant metastasis-free survival, (**d**) local recurrence-free survival, (**e**) regional recurrence-free survival and (**f**) locoregional recurrence-free survival

As shown in Fig. 1, none statistically significant survival difference was observed in pairwise comparison between groups. For the CV-IMRT arm, the 3-year OS, PFS, DMFS, LRFS, RRFS and LRRFS rates were 88.9, 84.4, 92.2, 91.1, 98.9 and 91.1%, respectively. For the RV-IMRT arm, the 3-year OS, PFS, DMFS, LRFS, RRFS and LRRFS rates were 92.2, 85.6, 90.0, 93.3, 98.9 and 93.3%, respectively. According to the subgroup analyses (Table 3), there was no significant survival difference between the CV-IMRT and RV-IMRT arms, irrespective of sex, age, T stage, N stage and clinical stage.

**Table 3:** Effects of subgroups on survival rates in nasopharyngeal carcinoma underwent CV-IMRT versus RV-IMRT

Variate		3-year OS			3-year PFS			3-year DMFS			3-year LRRFS		
		CV-IMRT	RV-IMRT	P	CV-IMRT	RV-IMRT	P	CV-IMRT	RV-IMRT	P	CV-IMRT	RV-IMRT	P
Sex	Female	91.7%	95.5%	1	91.7%	95.5%	1	100.0%	95.5%	0.478	91.7%	100.0%	0.49
	Male	87.9%	91.2%	0.583	81.8%	82.4%	1	89.4%	88.2%	1	90.9%	91.2%	1
Age	< 45	95.0%	90.0%	0.675	95.0%	80.0%	0.087	97.5%	85.0%	0.108	97.5%	90.0%	0.359
	≥45	84.0%	94.0%	0.2	76.0%	90.0%	0.108	88.0%	94.0%	0.487	86.0%	96.0%	0.16
T stage	T1–2	86.5%	94.0%	0.319	82.7%	88.0%	0.579	90.4%	94.0%	0.716	92.3%	94.0%	1
	T3–4	92.1%	90.0%	1	86.8%	82.5%	0.756	94.7%	85.0%	0.264	89.5%	92.5%	0.708
N stage	N0–1	97.4%	100.0%	1	94.7%	92.1%	1	97.4%	94.7%	1	97.4%	94.7%	1
	N2–3	82.7%	86.5%	0.787	76.9%	80.8%	0.811	88.5%	86.5%	1	86.5%	92.3%	0.526
Clinical stage	I-II	100.0%	100.0%	–	95.5%	95.5%	1	95.5%	100.0%	1	100.0%	95.5%	1
	III-IV	85.3%	89.7%	0.605	80.9%	82.4%	1	91.2%	86.8%	0.585	88.2%	92.6%	0.561

*OS* overall survival, *PFS* progression-free survival, *DMFS* distant metastasis-free survival, *LRRFS* locoregional recurrence-free survival, *CV-IMRT* conventional-volume intensity-modulated radiation therapy, *RV-IMRT* reduced-volume intensity-modulated radiation therapy

In multivariate Cox regression analyses, the predict value of various potential prognostic factors including gender, age, T stage, N stage, clinical stage, use of chemotherapy and radiotherapy pattern were evaluated (Table 4). Higher age was associated with high risk ratio for OS (RR = 2.408, 95% CI, 1.037–5.595; *P* = 0.041). Beyond that, no prognostic factor was significant for survival.

**Table 4:** Multivariate analysis of prognostic factors

Variate	OS		PFS		DMFS		LRRFS	
	RR(95% CI)	P	RR(95% CI)	P	RR(95% CI)	P	RR(95% CI)	P
Group								
RV-IMRT vs. CV-IMRT	0.580 (0.257–1.309)	0.19	0.826 (0.420–1.622)	0.578	1.285 (0.538–3.070)	0.573	0.553 (0.203–1.502)	0.245
Gender								
Male vs. Female	0.970 (0.382–2.464)	0.95	1.918 (0.736–4.998)	0.183	2.979 (0.684–12.978)	0.146	1.563 (0.439–5.562)	0.49
Age								
≥ 45 vs. < 45	2.408 (1.037–5.595)	0.041	1.451 (0.724–2.908)	0.294	1.053 (0.438–2.528)	0.909	1.607 (0.575–4.490)	0.365
T stage								
T3–4 vs. T1–2	0.817 (0.340–1.964)	0.651	0.916 (0.415–2.021)	0.828	1.068 (0.396–2.885)	0.896	1.179 (0.368–3.776)	0.781
N stage								
N2–3 vs. N0–1	2.455 (0.649–9.279)	0.186	2.192 (0.687–6.992)	0.185	2.715 (0.556–13.257)	0.217	1.490 (0.361–6.159)	0.581
Clinical stage								
III-IV vs. I-II	3.280 (0.281–38.226)	0.343	0.926 (0.183–4.683)	0.926	0.730 (0.080–6.639)	0.78	1.165 (0.129–10.480)	0.892
Neoadjuvant/adjuvant chemotherapy								
Yes vs. No	0.600 (0.085–4.238)	0.608	0.695 (0.108–4.464)	0.702	1.764 (0.084–36.960)	0.715	0.404 (0.047–3.457)	0.408
Concurrent chemotherapy								
Yes vs. No	0.987 (0.041–23.701)	0.994	3.338 (0.237–46.915)	0.371	0.796 (0.030–20.935)	0.891	–	–

*OS* overall survival, *PFS* progression-free survival, *DMFS* distant metastasis-free survival, *LRRFS* locoregional recurrence-free survival, *CV-IMRT* conventional-volume intensity-modulated radiation therapy, *RV-IMRT* reduced-volume intensity-modulated radiation therapy

### Toxicity

All 180 patients tolerated well and completed the planned treatment therapy. Table 5 showed the radiation toxicity profiles for both groups. With regard to late toxicities, nine patients had Grade 3/4 skin reaction and 13 patients suffered from Grade 3/4 hearing loss. Specially, two patients of the CV-IMRT group and one of the RV-IMRT group bore temporal lobe injury. One of the RV-IMRT developed second primary tumor. Overall, the RV-IMRT was associated with significantly reduced risk of late xerostomia (*P* = 0.039) and hearing loss (*P* = 0.008), compared with the CV-IMRT.

**Table 5:** Radiation toxicity profiles

Late toxicity	CV-IMRT(*n* = 90)					RV-IMRT(*n* = 90)					P
	Grade 0	Grade 1	Grade 2	Grade 3	Grade 4	Grade 0	Grade 1	Grade 2	Grade 3	Grade 4	
Xerostomia	12	60	18	0	0	20	60	10	0	0	0.039
Mucositis	16	64	10	0	0	20	62	8	0	0	0.406
Skin reaction	7	54	24	5	0	5	60	21	4	0	0.714
Dysphagia	12	70	8	0	0	15	69	6	0	0	0.432
Hearing loss	21	47	14	7	1	34	46	5	5	0	0.008
Blurred vision	81	8	1	0	0	84	4	2	0	0	0.441

*CV-IMRT* conventional-volume intensity-modulated radiation therapy, *RV-IMRT* reduced-volume intensity-modulated radiation therapy

In the subsequent subgroup analyses (Table 6), we tried to explore the potential population who might benefit from RV-IMRT with lower toxicity incidence. In terms of the radiation toxicity, the advantage of RV-IMRT was mainly observed in patients with T1–2 stage, N0–1 stage and I-II stage. For T1–2 stage individuals, significant lower rates of late xerostomia (P = 0.008) and hearing loss (*P* = 0.01) were noted in the RV-IMRT group than those in the CV-IMRT group.

**Table 6:** Effects of subgroups on late toxicity in nasopharyngeal carcinoma underwent CV-IMRT versus RV-IMRT

Variate		*P* value					
		Xerostomia	Mucositis	Skin reaction	Dysphagia	Hearing loss	Blurred vision
Sex	Female	0.511	0.562	0.362	0.36	0.077	0.625
	Male	0.038	0.145	0.893	0.716	0.042	0.532
Age	< 45	0.483	0.831	0.613	0.32	0.023	0.327
	≥45	0.036	0.384	0.215	0.838	0.12	0.756
T stage	T1–2	0.008	0.348	0.987	0.97	0.01	0.06
	T3–4	0.995	0.717	0.542	0.252	0.253	0.272
N stage	N0–1	0.255	0.546	0.676	0.787	0.01	0.969
	N2–3	0.079	0.553	0.81	0.236	0.185	0.252
Clinical stage	I-II	0.269	0.373	0.78	1	0.011	0.607
	III-IV	0.087	0.635	0.765	0.353	0.099	0.779
Neoadjuvant/adjuvant chemotherapy	No	0.715	0.897	0.363	0.675	0.017	1
	Yes	0.036	0.406	0.86	0.318	0.049	0.585
Concurrent chemotherapy	No	0.419	0.605	0.402	0.696	0.003	–
	Yes	0.053	0.492	0.831	0.318	0.069	0.412

*CV-IMRT* conventional-volume intensity-modulated radiation therapy, *RV-IMRT* reduced-volume intensity-modulated radiation therapy

## Discussion

Based on our study findings, generally speaking, the use of RV-IMRT for the treatment of nasopharyngeal carcinoma achieved similar treatment outcomes and did reduce the incidence of toxicity reactions compared versus CV-IMRT.

Since the early 1970s, traditional radiotherapy has been used for the treatment of NPC. The radiation technology is simple and imprecise, and radiation field encompassed in a two-dimensional portal is often large. Inevitably, various radiation-induced toxicities negatively affect patients’ quality of life. With the rapid development, IMRT has been effectively utilized in the treatment of NPC and has been regarded as a standard modality. This technique satisfies the possibility of improving survival rates and protecting the adjacent normal structures simultaneously [4–6]. Since IMRT and chemotherapy has obviously enhanced survival rates and lengthened survival time [7–10, 20–23], professors turn to the decrease of treatment toxicity and the improvement of patients’ quality of life. Yet, the delineation of clinical target volume in IMRT for nasopharyngeal carcinoma was largely derived from our experience of traditional radiotherapy, which is apparently improper in the precise radiation technology context. The dose coverage of the peripheral regions was suboptimal in the traditional radiotherapy era, however, isolated recurrence in those areas was rare [13–15]. In other words, the treatment volume that adjacent to the primary disease was excessive. Additionally, a variety of toxicities came along with. The reduction of the clinical target volume should be taken into account.

A study by Lin et al. [16] has reported 323 cases of non-metastatic NPC receiving IMRT using reduced clinical target volume. The definition of CTV in this reduced-volume IMRT protocol was substantially reduced when comparing with that in the RTOG protocol, as described in our study. With a median follow-up of 30 months, the 3-year LRFS, RRFS, DMFS, DFS and OS rates were 95, 98, 90, 85, and 90%, respectively. Besides, no Grade 3 or 4 xerostomia was detected beyond 3 months after the completion of treatment. In 2014, an update [24] of the reduced-volume IMRT analyzed 414 NPC patients: the 5-year LRFS, RRFS, DMFS, DFS and OS rates were 95, 97, 82, 77, and 80%, respectively. The survival results of these studies were comparative with that of other researches [25–27] used a relative large-volume IMRT by taking RTOG protocol as reference. This implied that using RV-IMRT in the treatment of NPC was safe and effective. Nevertheless, there has not been direct comparison between the two protocols.

In this study, we performed a retrospective analysis to compare the clinical treatment outcomes and toxicities of RV-IMRT with those of CV-IMRT for NPC patients. As for failure patterns, distant metastasis was the main failure mode with a rate of 11.7% in the whole population, locoregional recurrence accounted for 9.4% with the majority were in-field failures. None significant survival difference was shown between the two groups, irrespective of sex, age, T stage, N stage and clinical stage. Additionally, NPC patients who received RV-IMRT in our study had similar survival rates with those in the study by Lin et al. [16] (3-year OS, 92.2% vs. 90%, 3-year DMFS, 90.0% vs. 90%; 3-year LRFS, 93.3% vs. 95%; 3-year RRFS, 98.9% vs. 98%, respectively).

According to the univariate analyses, there were significant survival differences (OS, *P* = 0.007; PFS, *P* = 0.02) between different N category (N0–1 vs. N2–3). However, the multivariate analyses showed that neither T stage nor N stage was significant to predict survival outcomes. In addition, a number of papers [28–31] have also indicated that there was no significant survival difference between each T stage in NPC patients treated with IMRT. However, it should be noted that tumors were staged according to the clinical staging system based on data from conventional 2D-RT in these papers. We suspected that prognostic factors may vary with the progression of diagnostic and treatment techniques. Advanced imaging techniques can early detect occult metastases and accurately define the extent of tumor invasion. The application of IMRT and chemotherapy in the treatment of NPC has improved survival conditions obviously. As a consequence, the accuracy and applicability of staging systems should be reevaluated with the rapid development of imaging techniques and therapeutic methods. The latest eighth edition of the UICC/AJCC cancer staging classification was based on data in the IMRT era, which may perform better in predicting survival outcomes. Moreover, the multivariate analyses showed that receiving chemotherapy (concurrent chemotherapy, neoadjuvant or adjuvant chemotherapy) has no predictive value for treatment outcomes. Seemingly, it was not in line with our experience. We noticed that only a tiny proportion of patients included in this study have not received chemotherapy and the majority of which were presented with stage I. Early stage NPC cases treated with radiotherapy alone can be rendered disease-free in the long term. These could lead to the negative result.

Although a plenty of studies have substantiated that various radiation-induced toxicities are obviously reduced by using IMRT, the incidence of SPT is inverse. IMRT is likely to double the incidence of SPT compared versus 2D-RT from 1 to 1.75% for NPC patients [32, 33]. At the time of the last follow-up, however, only one patient (0.56%) developed SPT in our study. Overall, the RV-IMRT was associated with significantly reduced risk of late xerostomia (*P* = 0.039) and hearing loss (*P* = 0.008), compared versus the CV-IMRT. In the subgroup analyses, we tried to explore the potential population who might benefit from RV-IMRT. Generally, patients with T1–2 stage, N0–1 stage and I-II stage disease particularly benefit from RV-IMRT with similar survival rates and lower toxicity incidence. Hence, dosimetric improvements indeed translate into improvements in adverse events.

Despite of these satisfactory outcomes, the selection and definition of CTV in IMRT for NPC is far from addressed. Our study is supposed to has a certain guiding significance to the delineation of target volumes. Meanwhile, there are several limitations in our study. Firstly, the study was arranged as a retrospective trial with a small amount of patients. In spite of the well distributed patients, this study was performed in a nonendemic setting with relatively small amounts of cases. Furthermore, the small number of patients included may lead to an inadequate number of events needed for further analysis and limit the accuracy of the research results. Given all these, the generalization of the conclusions needs to be carefully considered, and well-designed trials are needed to confirm the findings in the future. Secondly, the median follow-up time was 50 months (range, 11–78 months) for the RV-IMRT arm. Since the majority of recurrence occurs in the first 2 years after the completion of radiotherapy [34–36], a median follow-up of 50 months signified that the true incidence of recurrence in the RV-IMRT arm may approximate our findings. With regard to the long term overall survival rate, longer follow-ups are in great need to evaluate the efficacy of RV-IMRT in the treatment of NPC. Thirdly, patients were paired using propensity score matching method to reduce imbalance between the experimental and control groups, thereby reducing the potential for bias. It should be noted that this method is of a few limitations. The weakness of PSM is that it does not consider the interaction between variables, but only focuses on the effect of a certain variable. Besides, after pruning some observations, the remaining samples may not be representative. Finally, the enrollment interval was longer than 10 years, many crucial factors such as the quality of image have varied during this period. The survival outcomes may be influenced by these factors.

## Conclusions

This study indicated that the use of RV-IMRT for the treatment of nasopharyngeal carcinoma did not adversely impact survival rates but did reduce the incidence of radiation toxicity compared versus the CV-IMRT. The delineation of target volumes in IMRT for nasopharyngeal carcinoma still needs to be optimized.

## Data Availability

The datasets used and analysed during the current study are available from the corresponding author on reasonable request.

## Abbreviations

2D-RT: Two-dimensional radiotherapy
AJCC: The American Joint Committee on Cancer
CT: Computed tomography
CTCAE: The Common Terminology Criteria for Adverse Events
CTV: Clinical target volume
CV-IMRT: Conventional-volume IMRT
DMFS: Distant metastasis-free survival
GTV: Gross tumor volume
IMRT: Intensity modulated radiation therapy
LN: Lymph node
LRFS: Local recurrence-free survival
LRRFS: Locoregional recurrence-free survival
MRI: Magnetic resonance imaging
NP: Nasopharynx
NPC: Nasopharyngeal carcinoma
OAR: Organ at risk
OS: Overall survival
PFS: Progression-free survival
PSM: Propensity score matching
PTV: Planning target volume
RRFS: Regional recurrence-free survival
RTOG: The Radiation Therapy Oncology Group
RV-IMRT: Reduced-volume IMRT
SPT: Second primary tumor
